# Coronary microvascular function in athletes with abnormal exercise test results

**DOI:** 10.1007/s12471-019-01336-6

**Published:** 2019-10-25

**Authors:** D. A. J. P. van de Sande, P. C. Barneveld, J. Hoogsteen, P. A. Doevendans, H. M. C. Kemps

**Affiliations:** 1 Department of Cardiology, Máxima Medical Center, Veldhoven, The Netherlands; 2 Department of Nuclear Medicine, Jeroen Bosch Hospital, ’s-Hertogenbosch, The Netherlands; 3 Department of Cardiology, University Medical Center Utrecht, Utrecht, The Netherlands; 4 Centraal Militair Hospitaal (CMH), Utrecht, The Netherlands; 5 Heart and Lungs, Netherlands Heart Institute (NHI), Utrecht, The Netherlands

**Keywords:** Athletes, Coronary microvascular function, Coronary flow reserve, Exercise testing

## Abstract

**Aims:**

In asymptomatic athletes, abnormal exercise test (ET) results have a poor positive predictive value. It is unknown whether abnormal ET results in the absence of obstructive coronary artery disease (CAD) are related to coronary microvascular dysfunction. It is also unknown whether they should be considered false-positive ET results or a consequence of physiological adaptation to sport. In our study, we evaluated whether athletes with abnormal ET results and documented myocardial ischaemia in the absence of obstructive CAD have an attenuated microvascular function and whether coronary microvascular dysfunction is related to endothelial dysfunction.

**Methods and results:**

Nine athletes with concordant abnormal ET and myocardial perfusion scintigraphy (MPS) results without obstructive CAD were compared with age- and gender-matched individuals with a low-to-intermediate a priori risk of CAD. Coronary flow reserve was assessed by Rubidium-82 positron emission tomography (PET) imaging. Endothelin‑1 concentrations were measured to evaluate endothelial function. Coronary flow reserve was significantly lower in athletes (3.3 ± 0.8 versus 4.2 ± 0.6, *p* = 0.014 respectively). Endothelin‑1 levels were significantly higher in athletes (1.3 ± 0.2 pg/ml versus 1.0 ± 0.2 pg/ml, *p* = 0.012 respectively). There was no correlation between endothelin‑1 concentrations and mean global coronary flow reserve (*r* = 0.12).

**Conclusion:**

Athletes with abnormal ET and MPS outcomes indicative for myocardial ischaemia and no obstructive CAD have a lower coronary flow reserve compared with non-athletes with low-to-intermediate a priori risk of CAD, suggesting an attenuated coronary microvascular function. Higher endothelin‑1 concentrations in athletes suggest that endothelial-dependent dysfunction is an important determinant of the attenuated microvascular function.

## What’s new

- Athletes with abnormal exercise testing and myocardial perfusion scintigraphy outcomes indicative for myocardial ischaemia in the absence of obstructive coronary artery disease have a lower coronary flow reserve compared with non-athletes with low-to-intermediate a priori risk of coronary artery disease.
- These findings suggest an attenuated coronary microvascular function.
- Higher endothelin‑1 concentrations in athletes suggest that endothelial-dependent dysfunction is an important determinant of the attenuated microvascular function.

## Introduction

The use of pre-participation screening in athletes to detect underlying cardiac diseases is recommended by the American Heart Association and the European Association of Cardiovascular Prevention and Rehabilitation [1, 2]. According to these guidelines, the use of exercise testing (ET) is restricted to individuals who show abnormalities suggestive for cardiac disease during primary screening or individuals with one or more traditional risk factors. However, in daily practice, many sporting events require athletes from every athletic level and age to perform an exercise test [3].

A problem associated with ET in asymptomatic athletes is its poor positive predictive value. In fact, a recent review demonstrated that obstructive coronary artery disease (CAD) was absent in up to 90% of the athletes with abnormal ET results [4]. Even when combined with myocardial perfusion scintigraphy (MPS), obstructive CAD was still absent in 80% of the athletes with concordant abnormal ET and MPS results [5].

Currently, it remains unknown whether concordant abnormal ET and MPS results in athletes should be considered false-positive test results, a consequence of physiological adaptation to sport, or a consequence of coronary microvascular dysfunction. Previous studies demonstrated an increased risk of major adverse cardiac events (MACE) in both non-athletic patients and athletes with abnormal ET and MPS results without obstructive CAD [5, 6], suggesting a pathophysiological basis of concordant abnormal test results. Moreover, results of animal studies suggested that coronary microvascular dysfunction may indeed play a role due to an inadequate increase in myocardial capillary density in response to development of training-induced myocardial remodelling/hypertrophy, remodelling of the intramural coronary arteries, autonomic imbalance or endothelial dysfunction [7, 8]. However, to date, no studies are available that address the coronary microvascular function in athletes with abnormal ET results. Therefore, the aim of the present study was to evaluate whether athletes with concordant abnormal ET and MPS results in the absence of obstructive CAD have an impaired coronary microvascular function as assessed by coronary flow reserve (CFR) and, furthermore, to evaluate whether coronary microvascular function was associated with endothelial dysfunction.

## Methods

### Study design and population

This study was designed as a single-centre prospective observational case-control study among asymptomatic recreational and competitive athletes who underwent pre-participation screening at the Department of Sports Medicine of the Máxima Medical Center and visited the department of Cardiology of the Máxima Medical Center. Athletes were defined as individuals who practise sports for at least 2.5 h a week for at least 30 weeks per year [9]. Asymptomatic athletes with an abnormal ET and abnormal MPS indicating myocardial ischaemia without obstructive CAD on coronary computed tomography angiography (CCTA) or coronary angiography within the last five years were selected from a previous study which enrolled 753 athletes. A total of 102 athletes (13.5%) showed abnormal ET results of which 51 underwent MPS. In the present study, the 9 athletes with abnormal ET and MPS results without obstructive CAD were included and underwent Rubidium-82 (^82^Rb) positron emission tomography (PET) imaging and CCTA in the Jeroen Bosch Hospital located in ’s-Hertogenbosch, the Netherlands. Prior to the ^82^Rb PET imaging and CCTA, the athletes underwent a direct venipuncture for the collection of blood samples (kidney function, lipid profile, endothelin‑1, glucose levels, C‑reactive protein and leucocyte count). After completion of the ^82^Rb-PET imaging, CCTA and venipuncture, all data of the athletes were interpreted and analysed. When data of ^82^Rb-PET imaging and CCTA were suitable for proper analysis, the athletes were matched according to gender (1:1) and age (years ± 2 years) with non-athletic individuals with a low-to-intermediate a priori risk of CAD according to the European Society of Cardiology (ESC) SCORE risk [10] and no obstructive CAD who already underwent ^82^Rb-PET imaging with low-dose CT and CCTA in the Jeroen Bosch Hospital. These individuals served as a control group and were selected via a digital query that only showed the electronic charts of individuals matching for age and gender who had undergone ^82^Rb-PET imaging in the last 2 years and in whom the scan was classified as normal (CFR > 2.0 and visual normal distribution of the tracer). Subsequently, the indications for ^82^Rb-PET imaging were evaluated. If imaging was used to rule out the presence of CAD in individuals with atypical complaints, the charts of these individuals were evaluated and ESC SCORE risk was calculated. From these, nine charts were randomly selected and included in the control group. The matched individuals underwent a direct venipuncture for the collection of the blood samples in order to assess endothelin‑1.

### ^82^Rb-PET imaging

All subjects were refrained from caffeine (at least 12 h prior to PET-imaging) and medication (theophylline, dipyridamole, asasantin, cafergot and/or ergotamine) was discontinued at least 48 h prior to PET imaging. The subjects were positioned in a 3-dimensional PET system (Siemens Biograph mCT 64 CT slices CT) and a low-dose CT scan was acquired for attenuation correction with an effective radiation dose of approximately 0.2–0.3 mSv. Subsequently, pharmacological stress was induced via administration of 140 mcg/kg/min adenosine for a 6-minute infusion duration. Three minutes after the start of infusion of adenosine, 1100 MBq of ^82^Rb-Chloride was injected. Dynamic stress images were obtained during a 7-minute acquisition after the ^82^Rb injection. After acquisition, the ^82^Rb-generator needed 10 min for regeneration and subsequently another 1,100 MBq ^82^Rb-Chloride was injected and rest images were obtained during 7 min of acquisition.

### CCTA

After PET imaging, subjects underwent CCTA (Siemens Flash dual source 128 slice). Prior to the ^82^Rb-PET scan and CCTA, a coronary artery calcium score (CACS) was measured. In case of a CACS 600 Agatston Units (AU), CCTA was cancelled as the diagnostic value of CCTA is markedly limited above this score. The effective radiation dose of CACS and CCTA was 2.5–3 mSv.

### Statistical analysis

All data were analysed using SPSS 22 statistical software (SPSS Inc, Chicago IL, USA). Differences in continuous data between both study groups were evaluated by the unpaired Student’s t‑test and categorical data by the chi-squared test. For all statistical comparisons, the level of significance was set at* p* < 0.05.

## Results

### Patient characteristics

In total, 9 asymptomatic male athletes with abnormal ET and MPS results and no obstructive CAD and 9 matched control subjects with a normal ^82^Rb-PET scan and CCTA and low-to-intermediate risk of CAD were included for analysis. Tab. 1 shows the characteristics of the athletes and the matched controls. The mean age of the athletes was 53.3 ± 11.3 years versus 52.7 ± 11.9 years (*p* = 0.905) in the control group. All subjects had a low ESC SCORE risk (2.6 ± 1.9% versus 2.4 ± 2.2%, *p* = 0.909 respectively) and showed comparable total cholesterol levels (4.8 ± 0.4 mmol/l versus 4.8 ± 0.6 mmol/l, *p* = 0.962). Resting systolic and diastolic blood pressure measurements were also comparable between both groups. Athletes achieved a mean maximal systolic blood pressure of 195.5 ± 21.9 mm Hg and mean maximal diastolic blood pressure of 77.8 ± 9.4 mm Hg. None of the athletes showed a hypertensive response to exercise. Resting ECG showed sinus rhythm in all subjects. Six athletes (67%) and 2 controls (22%) met ECG criteria (Sokolow) for left ventricular hypertrophy (*p* = 0.028), whereas incomplete right bundle branch block was found in 2 athletes (22%) and 4 controls (44%) (*p* = 0.470). T‑wave inversion beyond V2 was present in 1 athlete (11%) and 1 control (11%) (*p* = 1.000) and both subjects also showed incomplete right bundle branch block. Echocardiography was performed in 8 athletes and the mean left ventricular mass was 164.4 ± 29.1 g and left ventricular mass index 80.6 ± 14.7 g/m^2^. There was an inverse correlation between left ventricular mass index and CFR (*r* = −0.621).

**Table 1:** Comparison of the matched characteristics between athletes with concordant abnormal ET and MPS results and the matched individuals with low-to-intermediate risk of CAD

	Athletes (*n* = 9)	Controls (*n* = 9)
Age, years ± SD	53.3 ± 11.3	52.7 ± 11.9
Gender, M (%)	9 (100)	9 (100)
ESC SCORE RISK, mean (%)	2.6 ± 1.9	2.4 ± 2.2
Total cholesterol, mmol/l ± SD	4.8 ± 0.4	4.8 ± 0.6
*Resting blood pressure (mm Hg)*		
Systolic, mean ± SD	131.4 ± 19.6	123.4 ± 8.7
Diastolic, mean ± SD	66.8 ± 7.0	73.9 ± 11.0
*Maximal blood pressure (mm Hg)*		
Systolic, mean ± SD	195.6 ± 21.9	
Diastolic, mean ± SD	77.8 ± 9.4	
Rate-pressure product	9551.3 ± 1738.6	8723.6 ± 1457.5
Training hours/week	8 ± 2.7	n.a.
*Type of Sport*		
HS/HD, *n* (%)	8 (88.9)	n.a.
LS/HD, *n* (%)	1 (11.1)	n.a.
*Resting ECG*		
Sinus rhythm, *n* (%)	9 (100)	9 (100)
LVH	6 (66.7)	2 (22.2)
icRBBB	2 (22.2)	4 (44.4)
TWI	1 (11.1)	1 (11.1)
*Echocardiographic parameters*		
LVEDD, mean ± SD (mm)	48.7 ± 4.1	n.a.
IVSd, mean ± SD (mm)	9.8 ± 1.8	n.a.
PWTd, mean ± SD (mm)	9.4 ± 0.8	n.a.
LV mass, mean ± SD (g)	164.4 ± 29.1	n.a.
LV mass index, mean ± SD (g.m-2)	80.6 ± 14.7	n.a.
MPS ischaemic defects, mean ± SD	2.4 ± 0.7	n.a.
*Location ischaemic defects, N (%)*		
Anterior	4 (44.4)	n.a.
Anteroseptal	1 (11.1)	n.a.
Inferolateral	1 (11.1)	n.a.
Lateral	2 (22.2)	n.a.
Basal lateral	1 (11.1)	n.a.
*Location ischaemic defects on PET, N q(%)*		n.a.
Matched	4 (45)	
Mismatched	2 (22)	
No ischaemic defect	3 (33)	
*Primary diagnostic evaluation*		
CAG, normal	8 (88.9)	n.a.
CCTA, normal	1 (11.1)	n.a.

*ET* exercise test*, MPS* myocardial perfusion scintigraphy, *CAD* coronary artery disease, *SD* standard deviation,* ESC* European Society of Cardiology, *HS* high-static, *HD* high-dynamic, *LS* low-static, *LVH* left ventricular hypertrophy, *icRBBB* incomplete right bundle branch block, *TWI* T-wave inversion, *LVEDD* left ventricular end-diastolic diameter, *IVSd* interventricular septal wall thickness at diastole, *PWTd* posterior wall thickness at diastole, *LV* left ventricular, *MPS* myocardial perfusion scintigraphy, *CCTA* computed tomography coronary angiography, *CAG* coronary angiography, *n.a.* not applicable

Athletes practised predominantly high-static/high-dynamic sports (90%) categorised according to the Mitchell classification of sports for an average of 8.0 ± 2.7 training hours per week [11]. Using a 17-segment model of the left ventricle, an average of 2.4 ± 0.7 segments with reversible myocardial ischaemia were seen on MPS. The locations of these segments were anterior (44%), lateral (22%), anteroseptal (11%), inferolateral (11%) and basal lateral (11%). Resting and stress PET images were visually compared by the nuclear physician with the obtained perfusion images during MPS. PET imaging matched these ischaemic segments in 4 athletes (45%), there was a mismatch in 2 athletes (22%) and no ischaemic defects were seen in 3 athletes (33%). During primary diagnostic evaluation after abnormal MPS, 8 athletes underwent coronary angiography and 1 athlete underwent CCTA which showed no obstructive CAD in all athletes.

### Coronary blood flow measurements and coronary artery calcium scores

As presented in Tab. 2, low coronary artery calcium scores were observed in both groups (61.1 ± 157.5 AU versus 33.3 ± 50.0 AU, *p* = 0.621 respectively). Also, none of the included subjects showed obstructive CAD on CCTA. Resting and maximal myocardial blood flow were assessed via ^82^Rb-PET imaging in all three coronary artery territories. Global myocardial blood flow was calculated and corrected for rate pressure product. No significant differences were observed for (corrected) resting and maximal myocardial blood flow measurements between athletes and matched controls. However, CFR showed a significant lower mean global CFR in athletes (3.3 ± 0.8 versus 4.2 ± 0.6 in controls, *p* = 0.014 respectively), even when corrected for rate pressure product (3.2 ± 0.6 versus 3.9 ± 0.6 in controls, *p* = 0.045 respectively).

**Table 2:** Comparison of the ^82^Rb-PET imaging and CCTA results between athletes with concordant abnormal ET and MPS results and the matched individuals with low-to-intermediate risk of CAD

	Athletes (*N* = 9)	Control (*N* = 9)	*p*-value
*Resting coronary blood flow (ml/g/min)*			
LAD	1.1 ± 0.4	0.8 ± 0.2	0.052
RCx	1.0 ± 0.3	0.8 ± 0.2	0.143
RCA	0.9 ± 0.3	0.7 ± 0.2	0.104
Global	1.1 ± 0.3	0.8 ± 0.2	0.068
Corrected global flow	1.0 ± 0.2	0.9 ± 0.1	0.218
*Maximum coronary blood flow (ml/g/min)*			
LAD	3.5 ± 0.8	3.2 ± 0.6	0.349
RCx	3.0 ± 0.6	3.2 ± 0.4	0.262
RCA	3.1 ± 0.8	3.2 ± 0.5	0.741
Global	3.2 ± 0.7	3.2 ± 0.5	0.841
*Coronary flow reserve (ml/g/min)*			
LAD	3.3 ± 0.9	4.1 ± 0.7	0.080
RCx	3.1 ± 0.7	4.1 ± 0.5	0.003
RCA	3.4 ± 0.8	4.5 ± 0.6	0.004
Global	3.3 ± 0.8	4.2 ± 0.6	0.014
Corrected CFR	3.2 ± 0.6	3.9 ± 0.6	0.045
CACS	61.1 ± 157.5	33.3 ± 50.0	0.621
*CCTA*			
No epicardial CAD, *N* (%)	9 (100)	9 (100)	1.000

*PET* positron emission tomography,* ET* exercise test,* MP* myocardial perfusion scintigraphy,* CAD* coronary artery disease, *LAD* left anterior descending artery, *RCX* circumflex artery, *RCA* right coronary artery, *CFR* coronary flow reserve, *CACS* coronary artery calcium score, *CCTA* coronary computed tomography angiography

### Biochemical measurements

Endothelin‑1 concentrations were significantly higher in athletes (1.3 ± 0.2 pg/ml versus 1.0 ± 0.2 pg/ml, *p* = 0.012 respectively) as shown in Tab. 3. There was no correlation between endothelin‑1 concentrations and mean global CFR (*r* = 0.12). Total leucocyte count was significantly lower in athletes (5.5 ± 1.3 × 10^9/l versus 7.0 ± 0.7 × 10^9/l, *p* = 0.013 respectively). No significant differences were found in the levels of C‑reactive protein, creatinine clearance, glucose and lipid spectrum between groups.

**Table 3:** Comparison of the laboratory results between athletes with concordant abnormal ET and MPS results and the matched individuals with low-to-intermediate risk of CAD

	Athletes (*N* = 9)	Controls (*N* = 9)	*p-*value
CRP (mg/l ± SD)	0.9 ± 0.7	0.8 ± 0.6	0.624
Leukocyte count (x10^9/l ± SD)	5.5 ± 1.3	7 ± 0.7	0.013
Creatinine clearance (ml/min/1.7 ± SD)	57.1 ± 4.9	>60 ± 0.0	0.116
Glucose (mmol/l ± SD)	5.5 ± 0.8	5.5 ± 0.5	0.859
Total cholesterol (mmol/l ± SD)	4.8 ± 0.4	4.8 ± 0.6	0.962
Triglyceride (mmol/l ± SD)	1.1 ± 0.4	2.0 ± 1.4	0.103
HDL (mmol/l ± SD)	1.6 ± 0.4	1.4 ± 0.4	0.423
Chol/HDL ratio	3.2 ± 0.7	3.7 ± 1.3	0.383
LDL (mmol/l ± SD)	2.8 ± 0.4	2.5 ± 0.4	0.192
ET‑1 (pg/ml ± SD)	1.3 ± 0.2	1.0 ± 0.2	0.012

*ET* exercise test, *MPS* myocardial perfusion scintigraphy*, CAD* coronary artery disease, *SD* standard deviation, *CRP* C-reactive protein, *HDL* high-density lipoprotein cholesterol, *LDL* low-density lipoprotein cholesterol, *ET‑1* endothelin‑1

## Discussion

The results of this study demonstrate that athletes with abnormal ET and MPS outcomes indicative for myocardial ischaemia without obstructive CAD have a lower CFR when compared with non-athletes with low-to-intermediate a priori risk of CAD, suggesting an attenuated coronary microvascular function. Higher endothelin‑1 concentrations in athletes support the hypothesis that endothelial-dependent dysfunction is an important determinant of coronary microvascular function in these subjects.

CFR can be reduced by either stenoses of the epicardial coronary arteries, coronary microvascular dysfunction or a combination of both. As obstructive CAD was ruled out, the impaired CFR in the athletes included in the present study is likely to reflect coronary microvascular dysfunction. Although the global CFR exceeded the cut-off value of CFR < 2.0, which is associated with worse cardiovascular outcome [12], the CFR in these subjects was substantially lower when compared with other studies that investigated the CFR in athletes (3.3 ± 0.8 versus 5.9 ± 1.0 and 6.1 ± 1.9 respectively) [13, 14]. Even in non-athletes, the CFR values were higher when compared with CFR values of the athletes in the present study (3.8 ± 0.7 and 3.7 ± 0.7 versus 3.3 ± 0.8 respectively) [13, 14]. Although the previous studies used different modalities for measuring CFR (such as echocardiography and oxygen-15 labelled water [15O‑H_2_O]), in literature ^82^Rb-PET imaging has been validated by comparison with N13-ammonia and 15O‑H_2_O tracers with a high accuracy of myocardial blood flow estimates in the clinically relevant range. Therefore, the results of our study suggest an attenuated coronary microvascular function in athletes with abnormal ET and MPS results in the absence of obstructive CAD.

An attenuated coronary microvascular function in the absence of obstructive CAD reflects either functional or structural abnormalities of the small coronary arterioles as they are the major determinants of resistance in the coronary vasculature. A functional derangement that may cause a reduction in CFR is endothelial dysfunction [15]. An imbalance between the endothelial-derived vasodilator (nitric oxide) and vasoconstrictor (endothelin-1) is proposed to be the disrupted regulatory mechanism. During oxidative stress, such as exercise, there is overexpression of endothelin‑1 which leads to an enhanced vasoconstrictive state together with an impaired vasodilation through a reduced secretion of nitric oxide. In the present study, endothelin‑1 concentrations of the athletes were significantly higher when compared with matched individuals with a low-to-moderate a priori risk, suggesting an attenuated expression of endothelin‑1 in athletes. These results therefore suggest that endothelial-dependent dysfunction may be an important determinant of the attenuated coronary microvascular function in athletes with concordant abnormal ET and MPS results in the absence of obstructive CAD. This suggestion is strengthened by the observation that the expected sports-related increase in hyperaemic myocardial blood flow was not seen in this group of athletes. This indicates that there is a diminished vascular smooth muscle cell relaxation of the coronary arterioles due to the above-mentioned imbalance. Yet, besides functional derangements, structural abnormalities of the coronary microcirculation should also be considered.

Left ventricular pressure overload during exercise was shown to induce medial wall thickening of the arterioles with up to a twofold increase of the wall/lumen ratio [16], resulting in insufficient myocardial blood flow at rest. As animal studies showed that exercise-induced remodelling of the intramyocardial arterioles was highly age-dependent [7], this may have played a role in the present study targeting middle-aged athletes. Although no left ventricular hypertrophy was documented on echocardiography, data showed a significant inverse correlation between left ventricular mass index and CFR, supporting the theory that there was insufficient remodelling of the coronary microvasculature.

## Limitations

Before drawing conclusions, we need to address several limitations. First, the ^82^Rb-PET imaging results in athletes were compared with a non-athletic population with a low-to-moderate a priori risk who already underwent ^82^Rb-PET imaging, which could have led to a selection bias. Second, the study consisted solely of middle-aged male athletes with a low risk of CAD. Therefore, the results of this study cannot be generalised to athletes with a moderate or high a priori risk and women in whom an attenuated microvascular function could be more pronounced. Third, only athletes with segmental myocardial ischaemia on MPS were selected as coronary microvascular dysfunction could be distributed heterogeneously in the myocardium [17]. However, other studies showed that coronary microvascular dysfunction could also be distributed homogeneously throughout the walls of the left ventricle and is most frequently not associated with reversible perfusion defects seen on MPS [18]. Therefore, the results of the present study cannot be generalised for athletes with homogeneous coronary microvascular dysfunction and the prevalence of coronary microvascular dysfunction in athletes could be even higher than previously documented. Finally, as this study comprises only 9 athletes, studies in larger populations are needed to draw definitive conclusions.

## Conclusion

Athletes with abnormal ET and MPS results indicative for myocardial ischaemia and without obstructive CAD showed a lower CFR when compared with non-athletes with a low-to-intermediate a priori risk of CAD, suggesting an attenuated coronary microvascular function. Higher endothelin‑1 concentrations in athletes suggest that endothelial-dependent dysfunction may be an important determinant of the attenuated coronary microvascular function. Since this finding may have important clinical implications, future studies should be conducted with a long follow-up period to investigate whether or not preventive strategies should be considered.

## References

[1] Maron BJ, Araújo CG, Thompson PD (2001). Recommendations for preparticipation screening and the assessment of cardiovascular disease in masters athletes: an advisory for healthcare professionals from the working groups of the World Heart Federation, the International Federation of Sports Medicin. Circulation.

[2] Borjesson M, Urhausen A, Kouidi E (2011). Cardiovascular evaluation of middle-aged/senior individuals engaged in leisure-time sport activities: position stand from the sections of exercise physiology and sports cardiology of the European Association of Cardiovascular Prevention and Rehabilitation. Eur J Cardiovasc Prev Rehabil.

[3] Pigozzi F (2005). Role of exercise stress test in master athletes. Br J Sports Med.

[4] van de Sande DA, Breuer MA, Kemps HM (2016). Utility of exercise electrocardiography in pre-participation screening in asymptomatic athletes: a systematic review. Sport Med.

[5] van de Sande DA, Liem IH, Hoogsteen J (2017). The diagnostic accuracy of myocardial perfusion scintigraphy in athletes with abnormal exercise test results. Eur J Prev Cardiol.

[6] Hendel R, Abbott B, Bateman T (2011). The role of radionuclide myocardial perfusion imaging for asymptomatic individuals. J Nucl Cardiol.

[7] Duncker D, Bache R (2008). Regulation of coronary blood flow during exercise. Physiol Rev.

[8] Crea F, Camici PG, Bairey Merz CN (2014). Coronary microvascular dysfunction: an update. Eur Heart J.

[9] van Middelkoop M, van Linschoten R, Berger MY (2008). Knee complaints seen in general practice: active sport participants versus non-sport participants. BMC Musculoskelet Disord.

[10] Piepoli MF, Hoes AW, Agewall S (2016). European guidelines on cardiovascular disease prevention in clinical practice. Eur Heart J.

[11] Mitchell JH, Haskell W, Snell P (2005). Task force 8: classification of sports. J Am Coll Cardiol.

[12] Nakanishi K, Fukuda S, Shimada K (2013). Prognostic value of coronary flow reserve on long-term cardiovascular outcomes in patients with chronic kidney disease. Am J Cardiol.

[13] Hildick-Smith DJ (2000). Coronary flow reserve is supranormal in endurance athletes: an adenosine transthoracic echocardiographic study. Heart.

[14] Toraa M, Pouillard F, Merlet P (1999). Cardiac hypertrophy and coronary reserve in endurance athletes. Can J Appl Physiol.

[15] Montero D, Walther G, Diaz-Cañestro C (2015). Microvascular dilator function in athletes: a systematic review and meta-analysis. Med Sci Sports Exerc.

[16] Folkow B, Hallback M, Noresson E (1978). Vascular resistance and reactivity of the microcirculation in hypertension. Blood Vessels.

[17] Marroquin O, Holubkov R, Edmundowicz D (2003). Heterogeneity of microvascular dysfunction in women with chest pain not attributable to coronary artery disease: implications for clinical practice. Am Heart J.

[18] Kibel A, Selthofer-Relatic K, Drenjancevic I (2017). Coronary microvascular dysfunction in diabetes mellitus. J Int Med Res.

